# The Effects of Combined Treatments of Laser Engraving, Plasma Spraying and Resin Pre-Coating on Improving the Bonding Strength of Titanium Alloy and Carbon Fiber-Reinforced Polymer

**DOI:** 10.3390/polym16142041

**Published:** 2024-07-17

**Authors:** Wenyi Huang, Fei Cheng, Shihao Zuo, Yi Ji, Guangming Yang, Jiaxin He, Sidra Ashfaq, Yunsen Hu, Xiaozhi Hu

**Affiliations:** 1 Engineering Research Center of Biomass Materials, Ministry of Education, School of Materials and Chemistry, Southwest University of Science and Technology, Mianyang 621010, China; yiyi_iiid@163.com (W.H.); zsh13683403560@163.com (S.Z.); jiyi@swust.edu.cn (Y.J.); guangmingyang@mails.swust.edu.cn (G.Y.); hejiaxin2023@163.com (J.H.); sidriashfaq@gmail.com (S.A.); 2 Sichuan Sizhong Basalt Fiber Technology Research Co., Ltd., Basalt Fiber and Composite Key Laboratory of Sichuan Province, Dazhou 635756, China; 3 School of Mechanical Engineering, Jiangsu University of Science and Technology, Zhenjiang 212003, China; yshu1995@126.com; 4 School of Mechanical Engineering, University of Western Australia, Perth 6009, Australia; xiao.zhi.hu@uwa.edu.au

**Keywords:** hybrid bonding joint improving, laser engraving treatment (LET), resin pre-coating (RPC), CNT-reinforced epoxy pins (CREEP)

## Abstract

This study focused on effective methods of laser engraving treatment (LET), plasma spraying, and resin pre-coating (RPC) to manufacture the reinforced adhesive joints of titanium alloy and carbon fiber-reinforced polymer (TA-CFRP) composites. The combined treatments contributed to the creation of a better adhesive bonding condition and offer a vertical gap between circular protrusions to form epoxy pins and carbon nanotube (CNT)-reinforced epoxy pins. The bonding strength of the TA-CFRP composite was reinforced by 130.6% via treatments with a twice-engraving unit of 0.8 mm, plasma spraying, and RPC. The original debonding failure on the TA surface was changed into the cohesive failure of the epoxy adhesive and delamination-dominated failure of the CFRP panel. Overall, laser engraving has been confirmed as an effective and controllable treatment method to reinforce the bonding strength of the TA-CFRP joint combined with plasma spraying and RPC. It may be considered as an alternative in industry for manufacturing high-performance metal–CFRP composites.

## 1. Introduction

Carbon fiber-reinforced polymer (CFRP) is a high-performance composite consisting of carbon fibers and a polymer matrix [1,2,3], which is widely used in aerospace, automotive, sports equipment, construction, and medical equipment due to its light weight [4,5], high strength [6], and design flexibility [7]. Titanium alloy (TA) exhibits high strength, excellent corrosion resistance, and good biocompatibility, leading to its application in aerospace, automotive, medical implants, and marine environments. Pairing CFRP with TA to form composites can create a hybrid structure that sufficiently utilizes the characteristic of both materials [8,9].

Researchers have been investigating various ways to connect CFRP with TA, which include bolted joints [10], electroplating and brazing joining [11], adhesive joints [12], etc. The connection points of the bolts may increase the risks of crack extension and stress concentration, and this has an adverse influence on improving the joint strength. It is also noted that prolonged loading may lead to fatigue and loosening of the joints. The common welding treatments are likely to lead to the thermal degradation of CFRP due to the high temperatures. Adhesive bonding can avoid the drawbacks of these methods by applying an adhesive layer between the CFRP and the metal surface [13]. Epoxy resin is known for its high bonding strength, which allows for a strong and durable joint between the metal and CFRP [14,15]. This is especially beneficial in structural applications where load-bearing joints are critical.

The inadequate wettability and poor interfacial compatibility are the main concerns if common adhesive bonding is adopted between TA and CFRP, as they can lead to debonding failure, yielding a relatively low bonding strength. In order to improve the interlayer behaviors and properties of the composites, it is also necessary to modify the substrate surface before adhesive bonding. Laser engraving treatment (LET) is a high-efficiency and controllable method to prefabricate the designed surface conditions [16,17,18,19]. The main mechanism of LET is that it causes the TA surface form circular protrusions through the alternative processes of melting and solidifying. Those protrusions could not only create accessible spaces for the epoxy resin to construct the epoxy pins, but could also improve the wettability of the TA surface. LET has been employed to treat metal surfaces to improve the bonding properties of the composites in many publications. Lambiase et al. studied the effect of aluminum sheets’ laser-texturing on the shear strength of Al-Mg sheets: the polyvinyl chloride joints had an average strength of 16.1 MPa, higher than that of the untreated specimen [20]. Wu et al. found that the laser treatment of the metal surface could effectively improve the joint strength between carbon fiber-reinforced thermoplastic and 5052 aluminum. The maximum tensile shear force was over three times that of the untreated base [21]. The above-mentioned studies have proven that LET is successful in reinforcing the bonding joint, but the inadequate wetting and the void defects at the bottom of those protrusions also need to be avoided because they can deteriorate the bonding strength [22,23,24,25].

As is well-known to us, epoxy could not completely immerse into the cracks and grooves of the circular protrusions due to its high viscosity. Therefore, the special surface treatment technique should be further used to solve the aforementioned troubles and help create a stronger mechanical interlocking [26]. The resin pre-coating (RPC) technique is an effective method to reduce void defects and guide some additives into holes via the capillary forces through the volatilization of the solvent [27,28,29,30]. Liu et al. [31] used the RPC solution to perfectly fill micro-openings on the bamboo surface and the epoxy adhesive diffused well into the micro-openings to converge the residual epoxy (without hardener), while the interlocking effect between the substrate and the adhesive was enhanced, creating a stronger bonding joint. Cheng [32] discovered that the CNT-containing RPC solution could more easily enter the prepared micro-channels on the aluminum alloy surface and the CNT could be drafted into micro-channels with the rapid evaporation of acetone, which was very effective in reinforcing the bonding joint between dissimilar substrates. The plasma-spraying treatment will be very helpful in improving the physical and chemical bonds between the epoxy coating and porous oxide film before RPC is applied onto the TA surface.

In this study, LET was used to create the protrusions on the TA surface, while the plasma-spraying treatment and RPC were respectively adopted to form a better surface condition for adhesive bonding, and CNTs were introduced into holes to construct the CNT-reinforced epoxy-pins. The reinforced bonding joint of TA and CFRP was measured via a single-lap shear test, and their failure modes were investigated to discuss the reinforcing effect and mechanism.

## 2. Preparation and Characterization of Composites

### 2.1. Major Raw Materials and Manufacturing Equipment

The main raw materials used to prepare TA-CFRP composites included TA, CFRP, epoxy resin, carbon nanotubes, curing agent, and acetone, as shown in Table 1. Acetone acts as a dispersant for the epoxy resin, helping to obtain the RPC solution. The epoxy adhesive including epoxy resin and a hardener is used to bond TA and CFRP, and CNTs behave as the reinforcing additives in the RPC solution to strengthen the interface fiber bridging and establish a stronger bonding joint. The major manufacturing equipment are listed in Table 1.

### 2.2. Preparation of Composite Materials

**Figure 1:**
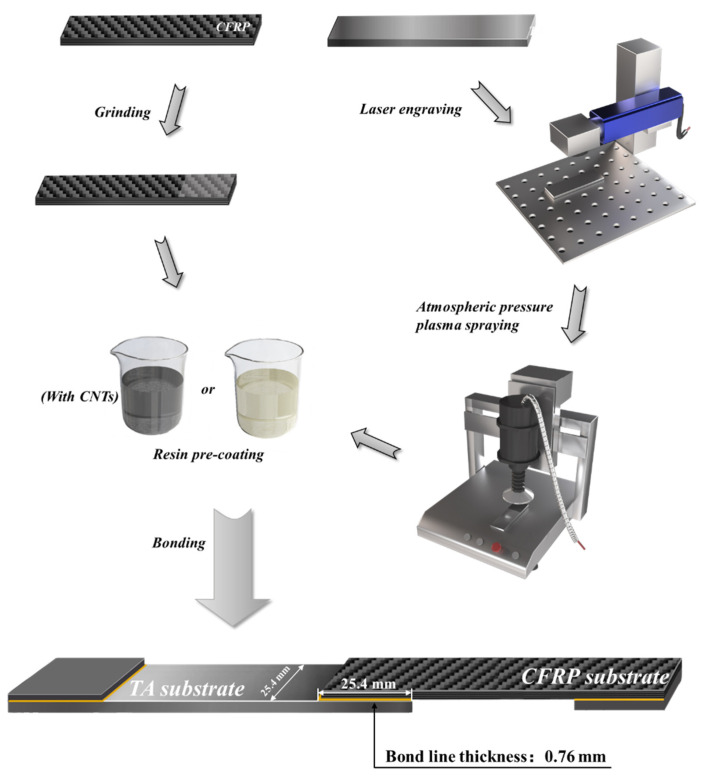
Schematic diagram of the surface treatment and adhesive bonding process of the TA-CFRP composite.

The preparation processes of TA-CFRP composites were designed as a substrate surface treatment, RPC treatment, and adhesive bonding, as shown in Figure 1.

LET and grinding were applied to TA and CFRP substrate surfaces, respectively. As-received TA substrates were placed in an acetone-filled beaker and ultrasonicated for 10 min to remove dust and impurities on the surface. Cleaned TAs were laid on the working table of the laser engraving machine for engraving. The parameters of LET were set to have a velocity of 80 mm/s, power of 24 W, and frequency of 30 kHz. The engraved pattern was a solid circle with various diameters of 0.4 mm, 0.6 mm, and 0.8 mm, and all the specimens were engraved once (LET1) and twice (LET2). Engraved TAs were ultrasonically cleaned by acetone for 10 min to remove surface dust, grease, and residues. The atmospheric pressure plasma-cleaning machine was further used to conduct plasma spraying on the TA surface for 10 s at a spraying distance of 10 mm, voltage of 220 V, and power of 20 W. CFRP substrates were ground using a DL6391 hand-held electric grinder for 10 s. The ground CFRP substates were then ultrasonically cleaned with acetone for 10 min.

TA and CFRP substrates underwent RPC treatments. Two RPC solutions were prepared: (1) 10 wt% epoxy resin (without hardener) and 90 wt% acetone; (2) 10 wt% epoxy resin (without hardener), 1 wt% CNTs and 89 wt% acetone. The engraved TA substrates were immersed in both a common RPC solution and CNT-reinforced RPC solution to form epoxy coatings and CNT-reinforced epoxy coatings on the substrate surface after the acetone evaporated. Ground CFRP substrates were only treated by common RPC solution to form epoxy coatings.

The above TA and CFRP substrates were bonded using Araldite^®^ AW106/HV953U two-component epoxy adhesive (mass ratio of epoxy and hardener equals 5:4). All the parameters of the adhesive bonding process complied with ASTM D5868, as illustrated in Figure 1. Bonded specimens were initially cured in situ at room temperature for 12 h, and then cured in a drying oven at 60 °C for 72 h to obtain the completely cured TA-CFRP composites. Table 2 demonstrated 13 groups of specimens with different engraved diameters and surface conditions.

### 2.3. Characterization Methods

The cross-section morphology of the TA with LET was tested using a metallographic microscope (WMJ-9590 metallographic microscope, Shanghai Wu Mor Optical Instrument Co., Ltd., Shanghai, China). The dimensions and characteristics of the circular protrusion on the engraved TA surface were observed.

The morphologies of the circular protrusions on the engraved TA surface and failed TA-CFRP composite were observed by scanning electron microscopy (SEM, Zeiss Ultra 55, Carl Zeiss GmbH, Göttingen, Germany). The elemental components in the selected areas were analyzed using equipped EDX.

The contact angles between the ultrapure water and TA surface with and without LET were detected by the Krüss DSA30 Droplet Shape Analyser (Krüs GmbH, Münster, Germany) The sessile drop method was used and the droplet size was 2.5 μL. Three measurements were conducted for each condition.

The single-lap shear test was carried out using a WANCE ETM105D Universal Testing Machine (Shenzhen WANCE Testing Machine Co., Ltd., Shenzhen, China). Tension was applied to the specimens at a constant rate of 2 mm/min and stopped once a rapid decline in load appeared. The bonding strength was calculated by dividing the maximum load by the area of the adhesive layer, and five specimens in each group were tested to ensure the reliability of the strength.

## 3. Results and Discussions

### 3.1. Surface Morphology of TA Substrates with LET

**Figure 2:**
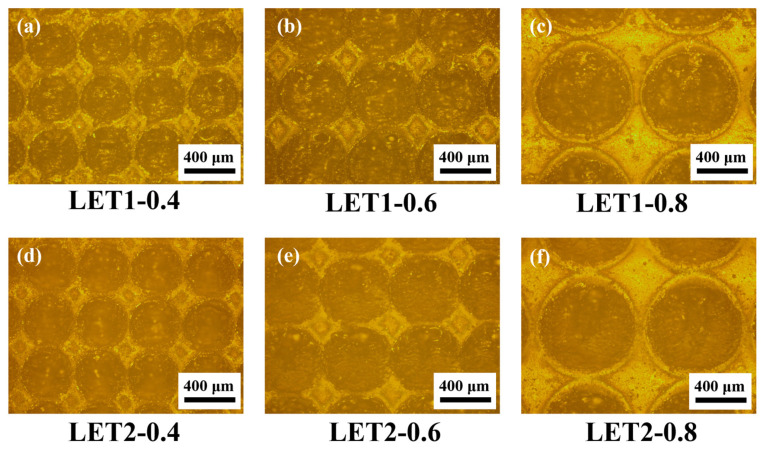
Surface images of the TA with different LET conditions. (**a**) LET1-0.4; (**b**) LET1-0.6; (**c**) LET1-0.8; (**d**) LET2-0.4; (**e**) LET2-0.6; (**f**) LET2-0.8.

Figure 2 shows the surface images of TA with different LETs conditions. The surface conditions of the specimens after being engraved once are displayed in Figure 2a–c and that of the specimens that were engraved twice are presented in Figure 2d–f. It can be seen that the diameters of the circular protrusions changed, with the engraving units increasing and grooves or channels forming between those circular protrusions, which provided the vertical space for the construction of epoxy pins on the TA surface.

**Figure 3:**
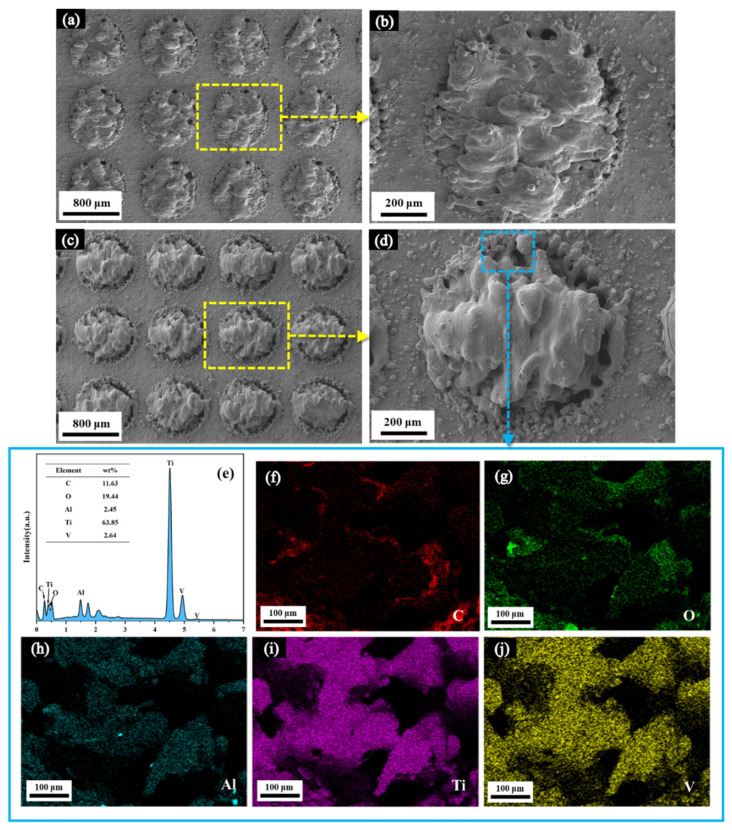
Representative SEM images of the engraved TA surface with the diameter of 0.8 mm: (**a**,**b**) once-engraved surface; (**c**,**d**) twice-engraved surface; (**e**–**j**) EDX spectroscopy of the highlighted region of circular protrusions on the twice-engraved surface of TA.

The representative SEM images of LET with a diameter of 0.8 mm and the related main elements are shown in Figure 3. Obvious circular protrusions could be observed on the engraved area, and some grooves or channels formed because of the uneven stacking during the cyclic melting and solidification process of the TA surface. The twice-engraved TA surface had more protuberant protrusions than the once-engraved TA surface in the treated circular region. These circular protrusions contributed to improving the contact area between epoxy and TA, even reinforcing mechanical interlocking once the epoxy adhesive totally filled the grooves or channels. The mass fractions of the main elements on the twice-engraved TA surface were C 11.63 wt%, O 19.44 wt%, Ti 63.85 wt%, V 2.64 wt%, and Al 2.45 wt%; the mass ratio of the O element significantly increased and that of Ti exhibited an over 20 wt% reduction compared to the as-received TA. This indicated that the circular protrusions might partially comprise the titanium oxide produced from the LET process. It should be stressed that distribution of these elements in Figure 3f–j is uneven and some dark regions can be observed; this is attributed to some cavities on the circular protrusion surface and was very difficult to detect.

### 3.2. Contact Angles of TA Surface under Different Conditions

**Figure 4:**
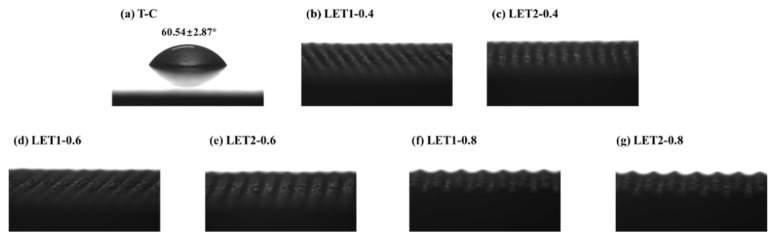
Contact angle test results of different TA surface conditions: (**a**) acetone ultrasonic cleaning, (**b**) LET1-0.4, (**c**) LET2-0.4, (**d**) LET1-0.6, (**e**) LET2-0.6, (**f**) LET1-0.8 and (**g**) LET2-0.8.

The results of the static contact angle tests on TA are shown in Figure 4, indicating the changes in the wettability of the TA surface before and after various LETs. The TA surface cleaned by acetone had a contact angle of 60.54 ± 2.87° (below 90°), demonstrating the good wetting of the surfaces. All the laser-engraved TA surfaces had extremely small contact angles (≈0°), which was because the droplets swiftly entered the gaps between the circular protrusions once they fell on these protrusions during the test, so the contact angle measurement was almost 0°. The circular protrusions could not only create a vertical space for the formation of an epoxy coating or CNT-reinforced epoxy coating, but also improved the wetting behavior of the TA surface, which contributed to the construction of epoxy pins on the bonding surface for stronger mechanical interlocking.

### 3.3. Surface Microstructure of TA after RPC Treatment

**Figure 5:**
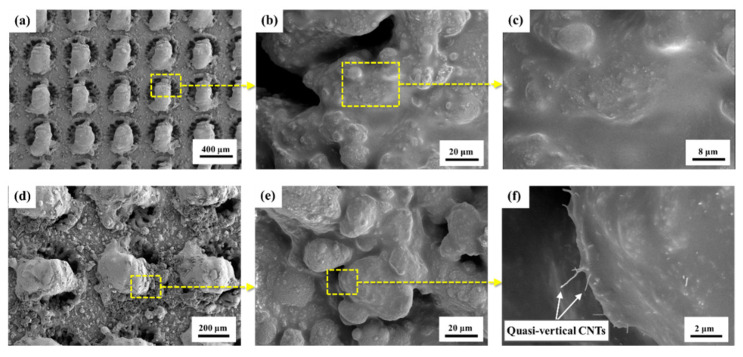
Representative SEM images of TA surfaces after RPC treatment: (**a**–**c**) surface of TA after RPC treatment; (**d**–**f**) surface of TA after RPC with CNT treatment.

The surface microstructure of TA after RPC (with CNTs) treatment is demonstrated in Figure 5. It can be observed that the pores on the circular protrusions were coated by a thin film of epoxy, with acetone evaporating totally, and the epoxy coatings were also formed on grooves and channels at a larger scale, as shown in Figure 5a–c. As shown in Figure 5e,f, CNTs were introduced successfully into gaps on the TA surface after RPC with CNTs was conducted, and they embedded quasi-vertically into the epoxy coatings on circular protrusions. It was also found that some CNTs laid in the epoxy coatings and were able to move via the diffusion between the epoxy adhesive (resin and hardener mixture) and the previously introduced epoxy (without hardener). This confirmed that the RPC (with CNTs) treatment was effective: both quasi-vertical CNTs and the laid CNTs contributed to the construction of an interfacial fiber bridging to strengthen the mechanical interlocking.

### 3.4. Bonding Strength Analysis of Specimens of Different Conditions

**Figure 6:**
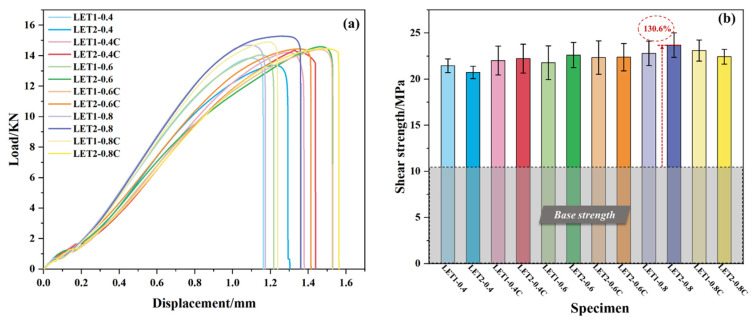
(**a**) Representative load–displacement curves of specimens with different treatments. (**b**) Average bonding strengths of each group (the error bars show the standard deviations in strength).

The load–displacement curves of the specimens after single-lap shear tests are shown in Figure 6a. All the curves were nonlinear at the beginning of the loading process owing to the initial gaps between the fixture components. The specimens then reached the elastic deformation stage as the load continuously increased and the curve linearly increase. The next nonlinear increase in the curve was due to the plastic deformation before peak loads occurred.

Figure 6b shows the bonding strength of different specimen groups. The bonding strength of the specimens cleaned only with acetone was 10.27 MPa (base strength highlighted by the gray region). The specimens with laser engraving, atmospheric pressure plasma-spraying, and RPC treatment had a higher bonding strength, and the LET2-0.8 specimens yielded the greatest strength of 23.69 MPa (130.6% higher than the base strength). It could be observed that the specimens engraved once or twice had a better bonding strength than the only acetone-cleaned specimens, implying that LET is an effective method to treat TA surfaces to obtain a stronger adhesive joint. The further introduction of CNTs contributed to toughening the epoxy layer and even the construction of fiber bridging, which improved the interfacial fiber behavior and adhesive bonding, as shown in Figure 6b.

### 3.5. Failure Mode Analysis

**Figure 7:**
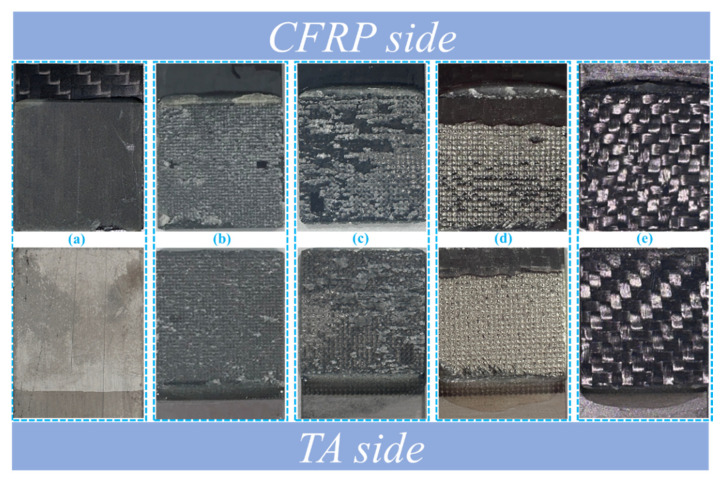
Typical failure appearances of bonding regions on TA and CFRP surfaces: (**a**) obvious de-bonding failure at the TA/epoxy interface; (**b**) cohesive-dominated failure of the epoxy adhesive layer; (**c**) cohesive-dominated failure of the epoxy adhesive layer; (**d**) peeled-off circular protrusions left on the CFRP side; (**e**) delamination-dominated failure of the CFRP panel.

**Figure 8:**
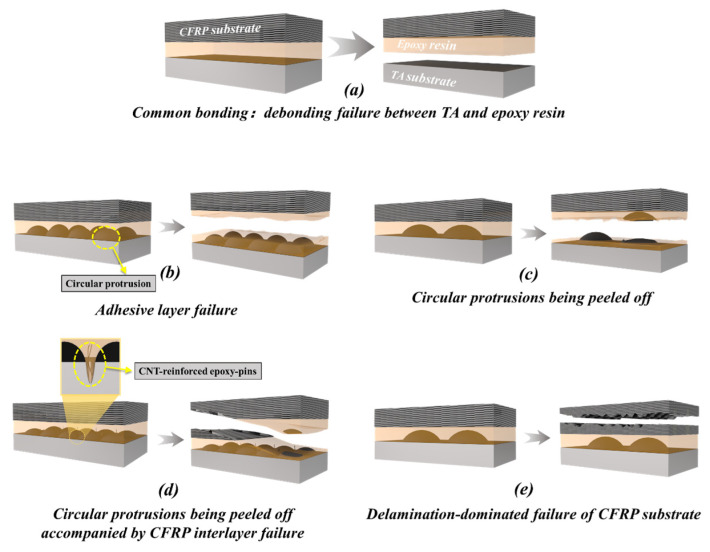
Schematic representation of failure modes of TA-CFRP composites after single-lap shear tests: (**a**) CFRP and TA ultrasonically cleaned with acetone; (**b**–**e**) grinding and RPC (or with CNTs) treatment of CFRP; laser engraving at different engraving diameters; RPC (or with CNTs) of TA.

Figure 7 shows the representative bonding region images of the TA-CFRP composite with different treatments after damage, and Figure 8 presents the failure mode schematic diagrams of TA-CFRP composites. It can be clearly seen that the adhesive layer de-bonded from the TA surface and remained on the CFRP side for only acetone-cleaned specimens owing to the poor compatibility between the TA and epoxy adhesive and the lack of mechanical interlocking; this was not good for the high-strength development of adhesive joints. Better bonding conditions were created after combination treatments of laser engraving, atmospheric pressure plasma-spraying, and RPC were adopted to improve the TA surface. The formed circular protrusions might create vertical spaces for the epoxy adhesive to access, leading to staggered epoxy pins being observed at the TA/epoxy interface instead of common bonding. The mechanical interlocking was strengthened and the cohesive failure of epoxy adhesive became dominated; this failure largely occurred in the engraved specimens, as shown in Figure 7b–d. If the bonding strength of the adhesive bonding joint including the adhesive layer, CFRP/epoxy interface, and TA/epoxy interface was great enough and even higher than the in-plane bonding strength of the laminated CFRP panels, the delamination-dominated failure of CFRP would be caused, as in Figure 7e.

**Figure 9:**
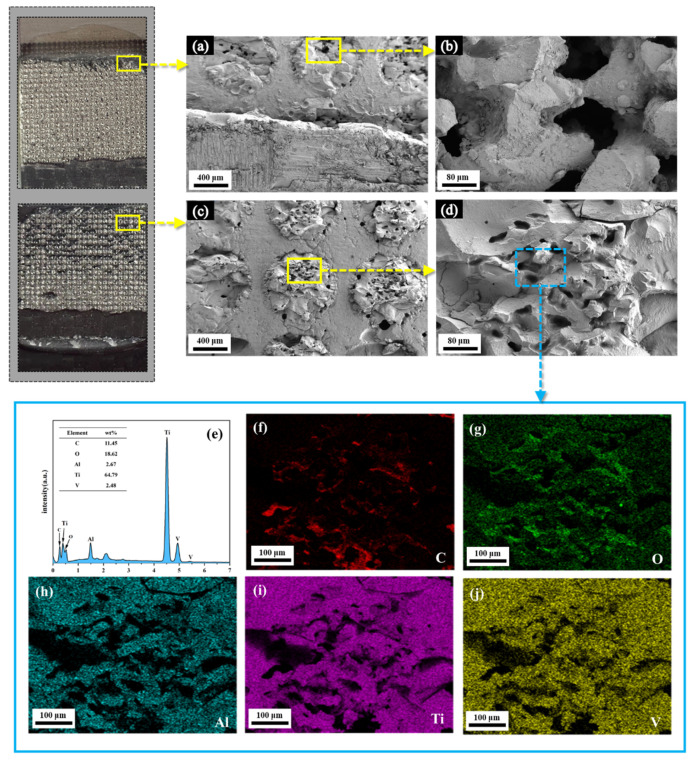
The surface pictures, SEM images, and EDX spectra of TA-CFRP specimens after failure: (**a**) and (**b**) SEM images of TA substrate side; (**c**,**d**) SEM images of CFRP substrate side. (**e**–**j**) EDX spectra and major element distributions of the highlighted region of circular protrusions left on the CFRP side.

The CNT-reinforced epoxy pins could be built in the gaps between circular protrusions, as depicted in Figure 8, and helped to strengthen the mechanical interlocking by toughening the epoxy adhesive and the fracture resistance of fiber bridging. This contributed to the formation of a stronger TA/epoxy interface and reinforced the adhesive bonding joint when the adhesive bonding strength was lower than the in-plane properties of the CFRP panel. Of course, it should be also stressed that the circular protrusions were likely to peel off under a relatively great external load as shown in Figure 9. The residues on the epoxy adhesive were confirmed to be circular protrusions through a comparison of the surface morphology and chemical component with the engraved specimens. This suggested that the circular protrusions obtained by LET were not too hard to break; they were not beneficial for the development of the bonding strength of the joint when the circular protrusions had greater heights and smaller engraving diameter units.

## 4. Conclusions

This study prepared reinforced adhesive bonding joints of TA-CFRP composites. LET, plasma-spraying, and RPC treatments were applied to the TA surface to obtain better adhesive bonding conditions. CNTs were introduced into the adhesive layer to form CNT-reinforced epoxy pins. The CFRP panels were ground and coated by epoxy coatings to compare their performances. Some prominent conclusions were emphasized, as follows.

(1) The strength of TA-CFRP-bonded joints was reinforced significantly by the united treatments of LET, plasma-spraying, and RPC treatments; the specimen with LET2-0.8 exhibited a 130.6% increment in bonding strength compared with the base.

(2) The original debonding failure on the TA surface was changed into the cohesive failure of the epoxy adhesive and delamination-dominated failure of the CFRP panel by prefabricating circular protrusions and forming epoxy pins or CNT-reinforced epoxy pins.

(3) Laser engraving was confirmed to be an efficient and controllable treatment method to reinforce the bonding strength of TA-CFRP joints. It may be considered as an alternative in industry for manufacturing high-performance metal–CFRP composites.

## Figures and Tables

**Table 1:** Special features and models of raw materials and equipment in this experiment.

Materials/Equipment	Special Features and Models	Origins
Titanium alloy	The compositions of the titanium alloy are 6.06 wt% Al, 4.03 wt% V, 0.15 wt% Fe, 0.17 wt% O, 0.02 wt% C, 0.02 wt% N, 0.015wt% H, and Ti balance (length 101.6 mm, width 25.4 mm, thickness 3.0 mm)	Wuxi Shengtai Technology Co., Ltd., Wuxi, China
Carbon fiber composite	3K twill weave carbon fiber panels (length 101.6 mm, width 25.4 mm, thickness 3.0 mm)	Carbonwiz Technology Co., Ltd., Shenzhen, China
Epoxy resin	Bisphenol A epichlorohydrin epoxy resin	Guangdong Advanced Chemical Materials Co., Ltd., Guangzhou, China
Hardener	Triethylenetetramine hardener	Guangdong Advanced Chemical Materials Co., Ltd., China
Acetone	AR 99.5%, boiling point 56 °C, toxic	Chengdu Kelong Chemical Ltd., Chengdu, China
Carbon nanotube	Helical multi-walled carbon nanotube (outer diameter 100–200 nm, length 1–10 μm)	XF Nano, Inc., Nanjing, China
Laser engraving machine	JL-F30	Liaocheng Jiuling Laser Equipment Co., Ltd., Liaocheng, China
Plasma cleaning machine	ZH-AP-500X-X	Shenzhen Zhenhua Plasma Intelligent Manufacturing Co., Ltd., Shenzhen, China
Electric grinder	DL6391, 120-grit,600-grit, 1000-grit aluminum oxide grinding wheel	Deli Group Co., Ltd., Ningbo, China

**Table 2:** Designed TA-CFRP composites with various laser engraving conditions.

Specimen Code	TA	CFRP	Specimen Number
T-C	Acetone cleaning	Acetone cleaning	5
LET1-0.4 ^a^	Laser engraving once + RPC	Grinding + RPC	5
LET2-0.4	Laser engraving twice + RPC	Grinding + RPC	5
LET1-0.4C ^b^	Laser engraving once + RPC (with CNTs)	Grinding + RPC (with CNTs)	5
LET2-0.4C	Laser engraving twice + RPC (with CNTs)	Grinding + RPC (with CNTs)	5
LET1-0.6	Laser engraving once + RPC	Grinding + RPC	5
LET2-0.6	Laser engraving twice + RPC	Grinding + RPC	5
LET1-0.6C	Laser engraving once + RPC (with CNTs)	Grinding + RPC (with CNTs)	5
LET2-0.6C	Laser engraving twice + RPC (with CNTs)	Grinding + RPC (with CNTs)	5
LET1-0.8	Laser engraving once + RPC	Grinding + RPC	5
LET2-0.8	Laser engraving twice + RPC	Grinding + RPC	5
LET1-0.8C	Laser engraving once + RPC (with CNTs)	Grinding + RPC (with CNTs)	5
LET2-0.8C	Laser engraving twice + RPC (with CNTs)	Grinding + RPC (with CNTs)	5

^a^ LET1-0.4: The specimen underwent laser engraving once and the laser-engraving diameter was 0.4 mm; RPC was then applied. ^b^ LET1-0.4C: The specimen underwent laser engraving once and the laser-engraving diameter was 0.4 mm; RPC with CNTs was then applied.

## Data Availability

Data are contained within the article.

## References

[1] Pramanik A., Basak A., Dong Y., Sarker P., Uddin M., Littlefair G., Dixit A., Chattopadhyaya S. (2017). Joining of carbon fibre reinforced polymer (CFRP) composites and aluminium alloys—A review. Compos. Part A Appl. Sci. Manuf..

[2] Backe S., Balle F. (2018). A novel short-time concept for fatigue life estimation of carbon (CFRP) and metal/carbon fiber reinforced polymer (MCFRP). Int. J. Fatigue.

[3] Xu M.-M., Huang G.-Y., Dong Y.-X., Feng S.-S. (2018). An experimental investigation into the high velocity penetration resistance of CFRP and CFRP/aluminium laminates. Compos. Struct..

[4] Zhou C., Li Y., Zhu G., Luo G., Zhao X., Yu Q. (2021). Tensile and flexural behavior of metal/CFRP hybrid laminated plates. Polym. Compos..

[5] Wang W., Chen X., Fan H. (2023). Modular technique to construct lightweight CFRP lattice structures. Thin-Wall Struct..

[6] Hosseini A., Ghafoori E., Motavalli M., Nussbaumer A., Zhao X.-L., Al-Mahaidi R., Terrasi G. (2019). Development of prestressed unbonded and bonded CFRP strengthening solutions for tensile metallic members. Eng. Struct..

[7] Jia Z.Y., Chen C., Wang F.J., Zhang C., Wang Q. (2020). Analytical model for delamination of CFRP during drilling of CFRP/metal stacks. Int. J. Adv. Manuf. Technol..

[8] Kolesnikov B., Herbeck L., Fink A. (2008). CFRP/titanium hybrid material for improving composite bolted joints. Compos. Struct..

[9] Ke L., Li C., He J., Shen Q., Liu Y., Jiao Y. (2020). Enhancing fatigue performance of damaged metallic structures by bonded CFRP patches considering temperature effects. Mater. Des..

[10] Camanho P., Fink A., Obst A., Pimenta S. (2009). Hybrid titanium–CFRP laminates for high-performance bolted joints. Compos. Part A Appl. Sci. Manuf..

[11] Guo K., Liu Y., Gou G., Zhang W., Gao W., Wang W. (2019). Electroplating and brazing joining of 5083 aluminum alloy to CFRP. Int. J. Mod. Phys. B.

[12] Kaiser I., Zhang C., Tan K.T. (2022). Mechanical behavior and failure mechanisms of CFRP and Titanium tubular adhesive lap joints at extreme temperatures. Compos. Struct..

[13] Raftery G.M., Harte A.M., Rodd P.D. (2009). Bonding of FRP materials to wood using thin epoxy gluelines. Int. J. Adhes. Adhes..

[14] Liu M., Rohde B.J., Krishnamoorti R., Robertson M.L., Dawood M. (2020). Bond behavior of epoxy resin–polydicyclopentadiene phase separated interpenetrating networks for adhering carbon fiber reinforced polymer to steel. Polym. Eng. Sci..

[15] Alwash D., Kalfat R., Du H., Al-Mahaidi R. (2021). Development of a new nano modified cement based adhesive for FRP strengthened RC members. Constr. Build. Mater..

[16] Vilhena L.M., Sedlaček M., Podgornik B., Vižintin J., Babnik A., Možina J. (2009). Surface texturing by pulsed Nd:YAG laser. Tribol. Int..

[17] Min J., Wan H., Carlson B.E., Lin J., Sun C. (2020). Application of laser ablation in adhesive bonding of metallic materials: A review. Opt. Laser Technol..

[18] Chen W., Lai W., Wang Y., Wang K., Lin S., Yen Y., Hocheng H., Chou T. (2015). Ultrafast Laser Engraving Method to Fabricate Gravure Plate for Printed Metal-Mesh Touch Panel. Micromachines.

[19] Nischkauer W., Vanhaecke F., Limbeck A. (2016). Self-aliquoting micro-grooves in combination with laser ablation-ICP-mass spectrometry for the analysis of challenging liquids: Quantification of lead in whole blood. Anal. Bioanal. Chem..

[20] Lambiase F., Paoletti A., Grossi V., Di Ilio A. (2017). Friction assisted joining of aluminum and PVC sheets. J. Manuf. Process.

[21] Wu L., Xiao B., Nagatsuka K., Nakata K., Ma Z. (2020). Achieving strong friction lap joints of carbon-fiber reinforced plastic and metals by modifying metal surface structure via laser-processing pretreatment. Compos. Struct..

[22] Xu Z., Yip W., Dong Z., Uddin M., Stevens G. (2024). On the laser surface pre-treatment to enhance the surface texture, wettability and adhesion bonding strength of aluminium 7075-T6 laminates. Compos. Interfaces.

[23] Yin H., Liu J., Xia H., Guo L., Ao X., Luo J., Yang Y. (2023). Effect of combination of microstructure and surface treatment on shear strength of precision bonded joints. J. Adhes..

[24] Wen L., Xu X., Qin L. (2023). Effect of Low-Temperature Plasma Surface Treatment on Bonding Properties of Single-Lap Joint of Thermosetting Composites. Polymers.

[25] Çoban O., Akman E., Bora M Ö., Genc Oztoprak B., Demir A. (2019). Laser surface treatment of CFRP composites for a better adhesive bonding owing to the mechanical interlocking mechanism. Polym. Compos..

[26] Palavra A., Coelho B.N., De Hosson J.T.M., Lima M.S.F., Carvalho S.M., Costa A.R. (2017). Laser surface treatment for enhanced titanium to carbon fiber-reinforced polymer adhesion. J. Braz. Soc. Mech. Sci. Eng..

[27] Zuo S., Cheng F., Yang G., Li J., Deng Y., Gou G., Cui X., Hu Y., Hu X. (2024). An effective micro-arc oxidation (MAO) treatment on aluminum alloy for stronger bonding joint with carbon fiber composites. Compos. Part A Appl. Sci. Manuf..

[28] Cheng F., Xu Y., Zhang J., Wang L., Zhang H., Wan Q., Li W., Wang L., Lv Z. (2023). Growing carbon nanotubes in-situ via chemical vapor deposition and resin pre-coating treatment on anodized Ti-6Al-4V titanium substrates for stronger adhesive bonding with carbon fiber composites. Surf. Coat. Technol..

[29] Hu Y., Zhang J., Wang L., Cheng F., Hu X. (2024). Enhancing adhesive bond strength of CFRP/titanium joints through NaOH anodising and resin pre-coating treatments with optimised anodising conditions. Chin. J. Aeronaut..

[30] Wang B., Ding G., Wang G., Kang S. (2020). Effects of resin pre-coating on interfacial bond strength and toughness of laminar CFRP with and without short aramid fibre toughening. J. Compos. Mater..

[31] Liu W., Xu H., Hu X., Yuan B., Tan B., Xu F. (2020). Strengthening and repairing of engineered bamboo-steel epoxy adhesive joints with carbon nanotube on the basis of resin pre-coating method. Eur. J. Wood Wood Prod..

[32] Cheng F., Hu Y., Lv Z., Chen G., Yuan B., Hu X., Huang Z. (2020). Directing helical CNT into chemically-etched micro-channels on aluminum substrate for strong adhesive bonding with carbon fiber composites. Compos. Part A Appl. Sci. Manuf..

